# Gene variants of the *SLC2A5* gene encoding GLUT5, the major fructose transporter, do not contribute to clinical presentation of acquired fructose malabsorption

**DOI:** 10.1186/s12876-022-02244-7

**Published:** 2022-04-06

**Authors:** Irina Taneva, Dorothee Grumann, Dietmar Schmidt, Elina Taneva, Ulrike von Arnim, Thomas Ansorge, Thomas Wex

**Affiliations:** 1 Department of Molecular Genetics, Medical Laboratory for Clinical Chemistry, Microbiology, Infectious Diseases and Genetics “Prof. Schenk/Dr. Ansorge and Colleagues”, Schwiesaustr. 11, 39124 Magdeburg, Germany; 2 Medical Office Internal Medicine and Gastroenterology, Olvenstedter Str. 11, 39108 Magdeburg, Germany; 3 Department of Gastroenterology, Hepatology and Infectious Diseases, Otto-Von-Guericke University, Leipziger Str. 44, 39120 Magdeburg, Germany

**Keywords:** Fructose malabsorption, SLC2A5, GLUT5, Promoter

## Abstract

**Background:**

While role of *ALDOB-*related gene variants for hereditary fructose intolerance is well established, contribution of gene variants for acquired fructose malabsorption (e.g. *SLC2A5*, GLUT5) is not well understood.

**Methods:**

Patients referred to fructose breath test were further selected to identify those having acquired fructose malabsorption. Molecular analysis of genomic DNA included (I) exclusion of 3 main *ALDOB* gene variants causing hereditary fructose intolerance and (II) sequencing analysis of *SLC2A5* gene comprising complete coding region, at least 20 bp of adjacent intronic regions and 700 bp of proximal promoter.

**Results:**

Among 494 patients, 35 individuals with acquired fructose malabsorption were identified based on pathological fructose-breath test and normal lactose-breath test. Thirty four of them (97%) had negative tissue anti-transglutaminase and/or deamidated gliadin antibodies in their medical records. Molecular analysis of *SLC2A5* gene of all 35 subjects identified 5 frequent and 5 singular gene variants mostly in noncoding regions (promoter and intron). Allele frequencies of gene variants were similar to those reported in public databases strongly implying that none of them was associated with acquired fructose malabsorption.

**Conclusions:**

Gene variants of coding exons, adjacent intronic regions and proximal promoter region of *SLC2A5* gene are unlikely to contribute to genetic predisposition of acquired fructose malabsorption.

## Introduction

Gastrointestinal symptoms are frequent causes for medical check-up in medical office and clinics as well. Pathophysiological mechanisms are numerous including infectious causes (viral, bacterial, parasitical), immunologically-related disorders (e.g. eosinophilic esophagitis, gastritis, coeliac disease, and chronic inflammatory bowel disease), intolerance towards nutrients or components (e.g. food-related allergies) and malabsorption syndromes of nutrients such as lactose [1] and fructose [2] as most common ones.

Malabsorption syndromes have rarely monogenic causes. Pathogenic mutations strongly affecting function of lactase [1, 3] or aldolase B [2, 4] as key enzymes for metabolizing lactose and fructose, respectively, are present in very few subjects. The overall majority of patients suffering from lactose- and fructose-related symptoms are caused multifactorially by hereditary, environmental, sociology-economic factors; in particular diet plays a major role. Lactose-related disorders had been widely studied for decades and have been comprehensively understood concerning its multi-factorial pathogenesis including genetic variants [5–7]. In contrast, fructose-related disorders are not well understood. Notably, fructose-associated disorders have sharply increased over the last 2 decades strongly associated with increasing demand of fructose consumption in a variety of food [8–10]. Incomplete intestinal absorption of fructose can lead to various symptoms in vulnerable subjects such as flatulence, diarrhea, bloating, nausea and pain [10–12]. Routinely, diagnosis of fructose malabsorption syndrome is mainly performed by hydrogen breath test after ingestion of defined amount of fructose [12]. Several studies demonstrated pathophysiological role of fructose transporters, in particular GLUT5, for the development of fructose-associated symptoms, although divergent results were reported. Mice experiments (either knockout or feeding models) highlighted a predominant role of *slc2a5* gene (glut5) as major intestinal transporter for fructose and signaling molecule for the induction of down-stream acting genes encoding fructolytic and gluconeogenic enzymes [13–15]. Furthermore, *slc2a5*-mRNA was found to be regulated by age and presence of fructose in rat model [16, 17]. In human intestinal cell line models fructose was shown to positively regulate *SLC2A5/GLUT5* expression by transcriptional and posttranscriptional regulation [18, 19]. However, ex vivo studies of human samples did not show differences in *SLC2A5*-mRNA or GLUT5-protein content in intestinal biopsies from patients with fructose intolerance [20]. In addition to GLUT5 in intestine, the glucose-transporter GLUT2 contributes to the hepatic uptake of fructose since GLUT5 is weakly expressed in liver [21]. Very recently, several studies revealed potential regulatory role of GLUT5 for malignancy and proliferation of various tumor cells driven by high consumption of fructose as carbon source [22, 23]. Based on these recent observations, diagnostic and therapeutic potential of targeting this metabolic pathway has been started to be investigated [24–27]. The only study addressing the role of gene variants of *SLC2A5*/GLUT5 for the development of fructose malabsorption studied 8 patients suffering from isolated fructose malabsorption without finding any functionally relevant gene variant. Mutational analysis was performed by single strand conformation polymorphism analysis and in one index case by Sanger sequencing [28]. In order to analyze potential role of *SLC2A5* gene variants in a larger cohort, a genomic region comprising the complete coding region, adjacent introns and 700 bp of proximal promoter region of 35 subjects with acquired fructose malabsorption were comprehensively analyzed by Sanger sequencing.

## Laboratory methods

### Study design and patients

**Fig. 1:**
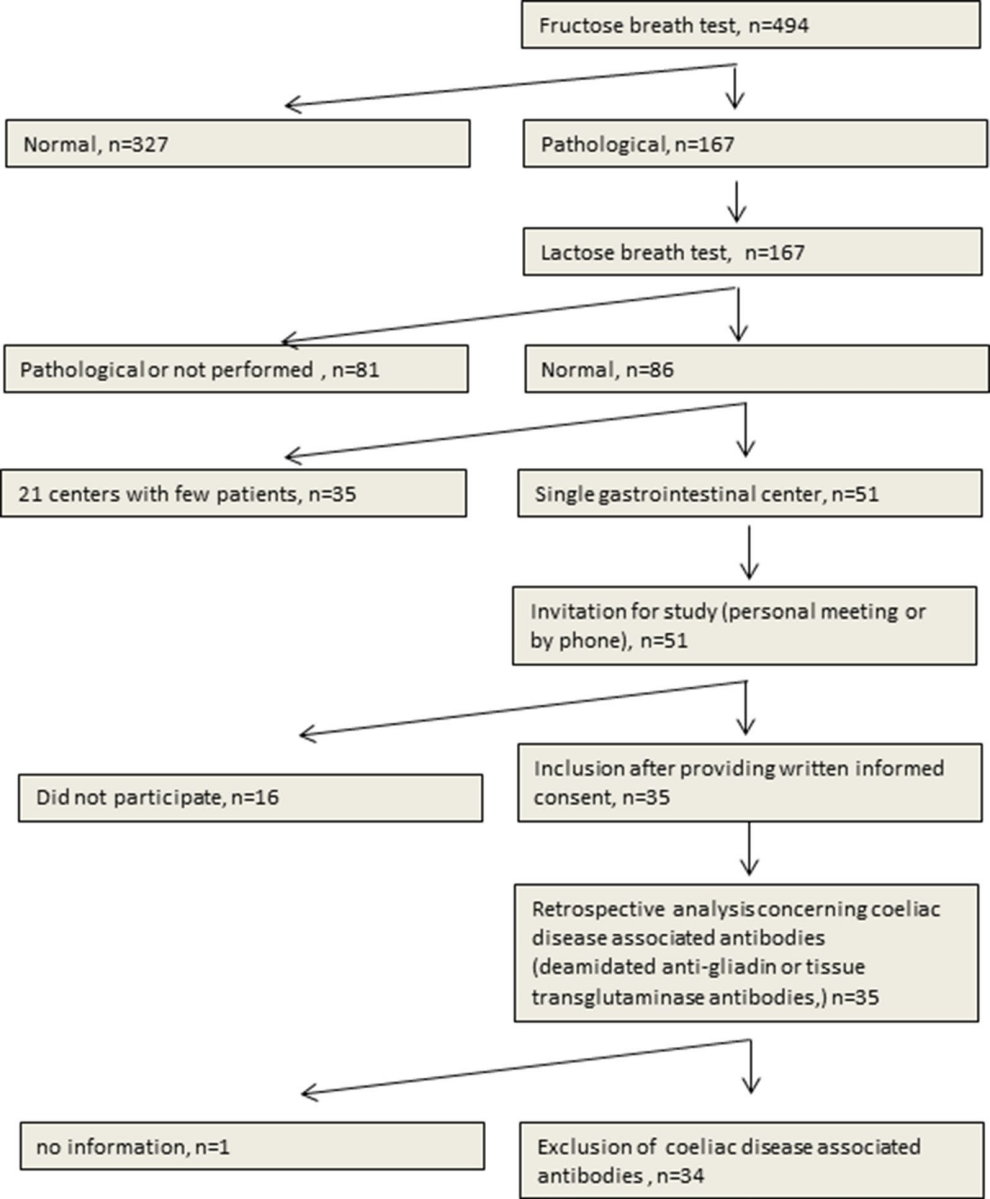
Study scheme

Study design was composed of retrospective and prospective parts (Fig. 1). Patients were included between 2013/01/01 and 2017/02/28. Patients referred to perform fructose-breath test in our center (n = 494) were screened for abnormal results suggesting malabsorption of fructose. Prior to the breath test, the 3 major gene variants of the ALDOB-gene causing hereditary fructose intolerance were excluded by PCR analysis. Among 167 patients with abnormal fructose breath test results, 86 presented normal results for lactose-breath test. Fifty-one patients from one gastroenterological center were selected allowing assessment of gastrointestinal symptoms by retrospective study of medical records. All 51 patients were invited to the study by phone and/or personal interview. Overall, 35 patients agreed participating to the study and provided signed informed consent. Study was performed in accordance with the Helsinki Declaration and the study and experimental protocols were approved by the Ethic Committee of the Land Sachsen-Anhalt (Vote No. 19/15).

Taken together, inclusion criteria were: (I) age > 18 years, (II) presence of symptoms implying upper gastrointestinal disorders (e.g. abdominal pain, diarrhea, vomiting, bloating, flatulence), (III) lack of 3 major gene variants of the *ALDOB*-gene causing hereditary fructose intolerance, (IV) pathological fructose-breath test and (V) normal lactose-breath test; details in Fig. 1. Note that no information in the medical records was available concerning the potential use of fructose–free diet in the study cohort. Based on the “pathological result” of the fructose breath test, which was a mandatory prerequisite for the study, a malabsorption of fructose after an intake of 25 g was suspected. Whether the patient was recommended to use/try fructose-free diet and the compliance of this aspect was not followed up in this study.

### Lactose-/fructose breath test

Breath tests were performed by routine methods. After 12 h fasting interval (not eating or drinking), patients were challenged with either 50 g lactose or 25 g fructose, dissolved in 200 ml water. Proportion of H_2_ in breath samples were analyzed every 30 min for a maximal period of 3 h. After identifying an increase of H_2_ samples were taken every 10–15 min. Generally, pathological findings for both breath tests were defined as increase of H_2_ content > 20 ppm compared to baseline (time point 0) and/or presence of gastrointestinal symptoms during test period. The presence of symptoms was assessed up to 8 h after test initiation by phone on the next day. For study purpose, pathological fructose-breath test was considered only if rise of hydrogen > 20 ppm was determined with and without presence of symptoms, while lactose breath test was also considered “positive” if only gastrointestinal symptoms appeared without increase of H_2_, since about 15% of all individuals are “H_2_-non-producer”.

### Molecular analysis of *ALDOB*-related SNPs and *SLC2A5* gene

Genomic DNA from blood samples were extracted from peripheral blood mononuclear cells using QIACUBE and corresponding DNA extraction kit (Qiagen, Hilden Germany).

Three aldolase B (*ALDOB*)-single nucleotide polymorphisms (SNPs) were genotyped by real-time polymerase chain reaction (RT-PCR) using TaqMan® assays with a 7500™ real-time cycler, in accordance with the manufacturer's instructions (Life Technologies, Carlsbad, California, USA) using Roche light cycler LCII. The 3 SNPs were: NM_000035.3(ALDOB):c.448G > C (p.Ala150Pro), rs1800546; c.524C > A (p.Ala175Asp), rs76917243 and c.1005C > G (p.Asn335Lys), rs78340951. Based on the absence of these SNPs, aldolase B deficiency could be excluded at > 90%.

Sequence analysis of *SLC2A5* included untranslated exon 1, all coding exons 2–13 with at least 20 bp of corresponding intron–exon boundaries and 700 bp promoter region upstream of untranslated exon 1. PCR products were amplified using Qiagen Hot Start Plus polymerase and M13/M13reverse-tagged primers and conditions as outlined in Table 1. Standard PCR conditions were: 1 × 94 °C, 5 min; 42 × (94 °C, 20 s; 54 °C, 30 s; 72 °C, 1 min) and 1 × 72 °C, 10 min.

**Table 1:** Primers used for amplification and sequencing

Region^a^	Sequence^b^	fragment size (bp)	Different conditions from standard protocol^d^
Promotor	M13-TCTCGCTCTGTCACCCA	456	60 °C + QS
	M13rev-GTCTTTGCCGTAGCCCA		
Promotor	M13-TAACAGTAACAGAAACGCTCC	431	60 °C
	M13rev-CCTAGTGGCTCAAAGATGG		
Promotor Exon 1 (UTR)^c^	M13-GGTCTTGCTCTGTCACCT	324	58 °C + QS
	M13rev-CCCTTCAGCTTCTGCCA		
Exon 2	M13-CCCACTTACTTAGCCAAACC	360	
	M13rev-TTCCCTCTGCAACACCA		
Exon 3	M13-TTGAGAAAGCCTGTAACCC	447	QS
	M13rev-CCCATCCCAAGAGACCT		
Exon 4	M13-CAGGTTATTTCATTGGGTGTC	339	
	M13rev-TGGTAAGGATTTCAGTTGTAGG		
Exon 5	M13-CCACACTGAGCGTATTCC	448	58 °C + QS
	M13rev-GTTTCACAGCAGAGGTATAGG		
Exon 6	M13-CCTTTGATCTGTTTCTCTTTCC	439	58 °C
	M13rev-AAAGTCCTGTCCTGTGGT		
Exon 7	M13-AAAGCTGTGCCCTCCTG	402	58 °C + QS
	M13rev-CCTTCTCTGCCTCATCCTC		
Exon 8	M13-TCTGCTGCCCTTCTTCC	574	QS
	M13rev-CATGACCACGTTCACGG		
Exon 9	M13-CGTGCTGAAGCTGTTCC	474	QS
	M13rev-CAGAGTTTCTGTAGTAGCGG		
Exon 10	M13-CTCAGGGTTGTGGGATTAGGA	640	QS
	M13rev-CAGACAAGCTAGGACGGGA		
Exon 11	M13-CATCTGCCTCATAGCCTG	602	58 °C + QS
	M13rev-CTCATTATGTGCCACCCA		
Exon 12, 13	M13-CCACATGCCCAAGAGTCCTG	730	58 °C + QS
	M13rev-AGCCCTTTGCACAGTTCCC		

^a^Numbering of exons is based on reference sequence *NG_050918.1*

^b^Sequencing tag M13 (gtaaaacgacggccagt) M13rev (ggaaacagctatgaccatg)

^c^Exon 1: untranslated region (UTR);

^d^Conditions refer to annealing temperature and addition of QS-solution (Qiagen, Hilden, Germany)

Sequence analysis was performed by standard Sanger sequencing protocols using GeXP platform as described by manufacturer (AbSciex, Darmstadt, Germany). Purification of PCR amplicons and sequencing products were performed using magnetic beads Agencourt AMPure XP and CleanSEQ (Beckman Coulter, Krefeld, Germany) as described by manufacturer. Sequence data were compared with reference sequences published at NCBI (NG_050918.1; NM_001328619.2) using CLC Workbench 8.23 (Qiagen, Hilden, Germany).

### Data presentation

Categorical data are expressed as absolute numbers with percentages. Age is shown as a mean with standard deviation. Frequencies of identified gene variants were compared to public databases such as “thousand genome project”. Due to the low numbers of (I) gene variants identified and (II) number of patients analyzed in the study, data are presented descriptively only and were not statistically analyzed.

## Results

### Characterization of study group

As summarized in Table 2, the majority of patients was female (29/35) and the mean age was about 38 years. Retrospective evaluation of clinical records revealed abdominal pain and diarrhea as leading symptoms. Data concerning the duration of symptomatic disease was available for 40% of the study group. Furthermore it was shown that 34 out of the 35 patients analyzed had negative serology for coeliac disease; either anti-deamidated gliadin IgG/IgA (n = 30) or anti-tissue transglutaminase IgG/IgA (n = 4) (Table 2).

**Table 2:** Demographic and clinical data of study group. Multiple symptoms were possible. Note that data were retrospectively recorded from medical records of patients; no structured interview was performed

Demographic parameter	Number/frequency
Gender (m/f)	6 (17%)/29 (83%)
Age (years); median (range)	36 (18–68)
*Symptoms*	
Abdominal pain	17 (48.6%)
Diarrhoea	12 (34.3%)
Meteorism	5 (14.3%)
Gastroesophageal reflux	5 (14.3%)
Irregular stool frequency	4 ((11.4)
Obstipation	3 (8.6%)
Haematochezia	2 (5.7%)
Not reported in detail	11 (31.4%)
*Onset of symptoms*	
Weeks to months	8 (22.9%)
Years	6 (17.1%)
Not recorded	21 (60.0%)
*Serological assessment of coeliac disease*	
Anti-deamidated gliadin IgG/IgA antibodies: negative	30/35 (85.7%)
Anti-tissue transglutaminase IgG/IgA: negative	4/35 (11.4%)
No information	1/35 (2.9%)

The routine follow up of patients included in the majority of cases upper gastrointestinal endoscopy (n = 25/35) and/or colonoscopy (n = 23/35). No malignancy was identified in the study cohort. Oesophago-gastroduodenoscopy revealed diagnoses such as gastritis, gastric erosions, gastroesophageal reflux disease, hiatal hernia, bulbitis or no alterations (n = 7). Note that none of the patients demonstrated signs of intestinal atrophy that is associated with coeliac disease. Colonoscopy revealed normal findings (n = 12) and diagnosis such as hemorrhoids, chronic sigmoiditis, proctitis, diverticolitis.

### Molecular analysis of *SLC2A5*

Sequence analysis of *SLC2A5* was successfully performed for all 35 patients. In total, 10 gene variants were identified from those 5 were frequent and 5 were identified just once (Table 3). The 5 frequent gene variants are located in promoter and adjacent intronic regions and demonstrated frequencies between 10.0 and 47.1%. Comparison of identified allele frequencies between public databases and own data demonstrate similar ranges for frequent variants (Table 3). The 5 rare variants (including the missense variant) were identified in individual patients only. Among them the only exonic variant found was a synonymous gene variant leaving amino acid p.Leu278 unchanged. Based on the criteria of the guidelines of the “American College of Medical Genetics” (ACMG) none of the gene variants are considered having pathological relevance (Table 3).

**Table 3:** Allele frequencies of GLUT5 gene variants in 35 patients with acquired fructose intolerance (TGP: Thousand Genome Project; https://www.internationalgenome.org/data/; gnomAD: Genome Aggregation Database; https://gnomad.broadinstitute.org/)

Gene variant (rs. No.)	NM_001328619.2 NP_001315548.1	Number of patients with gene variant (n = 35)	Allel frequency (%) own study/TGP-Europe/gnomAD-Europe)	Classification based on ACMG-guidelines (www.varsome.com)
rs958806131	c.-269-247 C>T	1	1.4/n.d./0.01	VUS3
rs1705285	c.-269-213 T>C	22	34.3/39.0/36.1	Benign
rs12117043	c.-269-202 C>T	22	34.3/32.2/30.8	Benign
rs35276984	c.-269-135 ins T	31	47.1/59.0/59.4	Benign
rs5438	c.-25 G>A	1	1.4/5.8/5.6	VUS3
rs3737661	c.294-56 C>A	7	10.0/5.1/n.d	Likely benign
rs139477702	c.832 C>T, p.Leu278=	1	1.4/0.2/0.3	Likely benign
rs11121306	c.1098+145 C>T	19	28.6/27.4/26.3	Benign
rs370588099	c.1175-38 G>A	1	1.4/n.d./0.01	VUS3
unknown	c.1302+21 A>C	1	1.4/n.d./n.d	Unknown

## Discussion

Here, we demonstrated that gene variants of *SLC2A5* gene encoding the fructose transporter GLUT5 are not generally involved in the pathogenesis of acquired fructose malabsorption. Based on the number of subjects studied (n = 35) we cannot exclude a rare role of such gene variants, but the portion of patients suffering from “pathogenic GLUT5 variants” is likely to be < 3–5%.

A major strength of the study is the clinically based definition of the study cohort prior to molecular analysis. Related gastrointestinal disorders such as hereditary lactose-intolerance and acquired lactose malabsorption, both leading to pathological lactose breath test, were excluded. Hereditary ALDOB deficiency was practically ruled out by analyzing the 3 major mutations of *ALDOB* leading to this disease. Coeliac disease, also considered as chimera among gastrointestinal diseases, was kept out of the study group by 2 approaches. First, since secondary lactose malabsorption is a well-known leading symptom for coeliac disease, these patients were excluded by abnormal lactose breath test. Second, for 34 out of the 35 individuals, corresponding serological parameters (anti-tissue anti-transglutaminase or anti-deamidated gliadin antibodies) were found to be negative.

The symptoms reported by our patients are rather unspecific and in line with those reported in similar studies with patients (I) suffering from fructose malabsorption tested by breath test [29–31], or (II) classified as having irritable bowel disease (IBS) [32, 33]. However it is notable that symptoms in our studies were assessed retrospectively only by analyzing patients’ medical records, and no structured interview or assessment of questionnaire in context to e.g. IBS-related Rome criteria [34] was performed.

In summary, the 35 subjects included in the molecular analysis of the *SLC2A5* gene presented (I) clinically relevant symptoms that are consistent with acquired fructose-malabsorption, (II) demonstrated abnormal fructose-induced breath test and (III) relevant other related diagnoses (e.g. coeliac disease, hereditary fructose intolerance) were basically excluded. Taken together, we are confident that overall the great majority of these 35 subjects are patients suffering from acquired fructose malabsorption. It has been shown that the individual ability of metabolizing fructose for subjects without any side effects differs widely from 5–50 g (reviewed in [21]) supporting the multi-factorial etiology of acquired fructose malabsorption.

The aim of the study, molecular analysis of *SLC2A5* gene concerning gene variants associated with fructose malabsorption, was based on other studies showing the role of SNPs/mutations affecting the uptake/metabolism of related sugars. Variants including partial deficiency of sucrose-isomaltase were shown to be associated with IBS [35]. Analysis of UK biobank data revealed that gene variants in human ketohexokinase gene are associated with loss of function and resulting in the rare benign condition of fructosuria [36]. In vitro mutation analysis in rats between GLUT5 and its closest related transporter (GLUT7) revealed that single amino acids (e.g. p.Gln166Glu) are responsible for the specific transport of fructose, and mutation of this residue to p.166Glu results in the uptake of glucose, whereas other variants and chimera between GLUT5 and GLUT7 demonstrated strong reduction or even complete lack of fructose uptake [15].

The fact that the allele frequencies of the 10 gene variants between ours and those reported in database were very similar strongly implies that none of these variants have a relevant role for the clinical manifestation of acquired fructose malabsorption. Notably, 5 of the 10 gene variants were singular findings that do not allow any general conclusion due to study size. But taken into account the very low frequencies reported in databases, the potential relevance for the very frequent fructose-absorption syndrome seems to be very limited. Since the study was considered as pilot study to identify potential pathogenic gene variants of the *SLC2A5* gene in relation to acquired fructose malabsorption, we did not include an own control group in the study design and decided instead to initially compare identified frequencies of gene variants with public databases.

While this study demonstrate that *SLC2A5*-related gene variants do not play a relevant role in the pathogenesis of acquired fructose malabsorption, other pathogenic factors have been recently identified to be associated with this disorder. Trelis and co-workers identified a frequent association of the disease with the infection of parasites, in particular Giardia intestinalis [37]. Several animal studies identified specific changes in the gut microbiome in context to genetic host factors [38] and the intake of fructose [38, 39] showing that Akkermansia spec. seems to play an important role in the prevention fructose-induced metabolic dysregulation. Over-expression of *slc2a5* in *slc2a5*/glut5-knock out mice let to profound increase of fructose utilization and subsequent higher levels of Clostridium and Enterococcus spec. [40]. Overall most related studies demonstrate that higher intestinal luminal levels of fructose caused by changes in fructose consumption or absorption will likely affect bacterial load and composition of the microbiome (reviewed in [41]).

In humans, several studies highlighted the role of the transcription factor ChREBP encoded by the MLXIPL gene for the predisposition concerning fructose intolerance malabsorption [21, 42] and diarrhea—predominant IBS patients with impaired intestinal fructose transport [43]. The association between ChREBP and fructose malabsorption was further supported by animal models [44, 45]. Nuclear receptor LXR (lxralpha, NR1H3) is another transcriptional regulator of GLUT5 expression identified in mice and human that is thought to be a potential pharmaceutical target for selective modulation of GLUT5 expression in context to cancer and metabolic disease [46]. Notably, authors identified a functional LXR responsive element in the human *SLC2A5* promoter region located at position-385 based on transcriptional start site, but none of our 35 patients showed a variation at this position.

Overall, these different findings strengthen the hypothesis that fructose-related malabsorption syndrome associated with different pathological conditions has multi-factorial etiology. Different transcriptional regulatory patterns affecting the *SLC2A5* gene expression contribute to the pathology, whereas gene variants of *SLC2A5* including the promoter region, which was the focus of this study, do not play a relevant role.

## Data Availability

All data generated or analysed during this study are included in this published article.
